# A Systematic Literature Review of 3D Deep Learning Techniques in Computed Tomography Reconstruction

**DOI:** 10.3390/tomography9060169

**Published:** 2023-12-05

**Authors:** Hameedur Rahman, Abdur Rehman Khan, Touseef Sadiq, Ashfaq Hussain Farooqi, Inam Ullah Khan, Wei Hong Lim

**Affiliations:** 1 Department of Computer Games Development, Faculty of Computing & AI, Air University, E9, Islamabad 44000, Pakistan; hameed.rahman@mail.au.edu.pk; 2 Department of Creative Technologies, Faculty of Computing & AI, Air University, E9, Islamabad 44000, Pakistan; abdurrehamn809@gmail.com; 3 Centre for Artificial Intelligence Research, Department of Information and Communication Technology, University of Agder, Jon Lilletuns vei 9, 4879 Grimstad, Norway; 4 Department of Computer Science, Faculty of Computing AI, Air University, Islamabad 44000, Pakistan; ashfaq.hussain@mail.au.edu.pk; 5 Department of Electronic Engineering, School of Engineering & Applied Sciences (SEAS), Isra University, Islamabad Campus, Islamabad 44000, Pakistan; inamullahkhan05@gmail.com; 6 Faculty of Engineering, Technology and Built Environment, UCSI University, Kuala Lumpur 56000, Malaysia; limwh@ucsiuniversity.edu.my

**Keywords:** 3D deep learning (3DDL), computed tomography (CT) reconstruction, systematic literature review

## Abstract

Computed tomography (CT) is used in a wide range of medical imaging diagnoses. However, the reconstruction of CT images from raw projection data is inherently complex and is subject to artifacts and noise, which compromises image quality and accuracy. In order to address these challenges, deep learning developments have the potential to improve the reconstruction of computed tomography images. In this regard, our research aim is to determine the techniques that are used for 3D deep learning in CT reconstruction and to identify the training and validation datasets that are accessible. This research was performed on five databases. After a careful assessment of each record based on the objective and scope of the study, we selected 60 research articles for this review. This systematic literature review revealed that convolutional neural networks (CNNs), 3D convolutional neural networks (3D CNNs), and deep learning reconstruction (DLR) were the most suitable deep learning algorithms for CT reconstruction. Additionally, two major datasets appropriate for training and developing deep learning systems were identified: 2016 NIH-AAPM-Mayo and MSCT. These datasets are important resources for the creation and assessment of CT reconstruction models. According to the results, 3D deep learning may increase the effectiveness of CT image reconstruction, boost image quality, and lower radiation exposure. By using these deep learning approaches, CT image reconstruction may be made more precise and effective, improving patient outcomes, diagnostic accuracy, and healthcare system productivity.

## 1. Introduction

**Figure 1:**
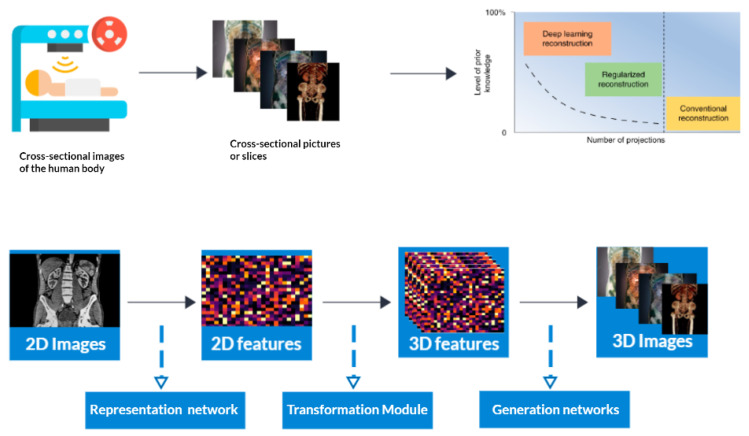
3D Reconstruction and enhanced visualization: CT scan data transformed into a detailed 3D image using deep learning techniques.

Computed tomography (CT) is a medical imaging method that offers cross-sectional images of the body of a person, which allows for the visualization of organs and tissues aiding in the diagnosis and management of a wide range of medical conditions, including cancer [1,2], heart disease [3], and neurological disorders [4]. CT refers to a computerized X-ray imaging procedure. In this method, a patient is swiftly rotated around a narrow beam, creating signals processed by the machine’s computer to produce cross-sectional pictures or slices. Once the scanner has gathered a number of these slices, which are known as tomography pictures, they are stacked together to create three-dimensional representations of the patient [5,6,7,8,9]. These three-dimensional representations are created by applying an algorithm to the raw data, resulting in image slices that are then reconstructed into a 3D volume [10,11]. This process is called CT reconstruction (Figure 1). Figure 1 is attributed to the work by Liyue Shen in his paper titled ‘Patient-specific reconstruction of volumetric computed tomography images from a single projection view via deep learning’ [12].

Computed tomography (CT) initially relied on traditional computationally intensive analytical techniques used in image reconstruction, such as filtered back-projection (FBP). However, these methods often had limitations, including image noise and other artifacts that compromised the quality and efficiency of the reconstructed images. To address these challenges deep learning emerged as a powerful tool in CT image reconstruction. By leveraging deep neural networks, deep learning algorithms have significantly improved the quality and efficiency of CT image reconstruction.

In addition to 2D image reconstruction, deep learning has also been extended to 3D image reconstruction in CT. By incorporating volumetric information, deep learning algorithms can generate more precise and detailed 3D images, further enhancing the diagnostic capabilities of CT scans [13,14,15].

What exactly is 3D deep learning? It is a sort of machine learning that examines and interprets 3D data using AI neural networks. It requires preparing neural networks to discover intricate correlations and characteristics in 3D datasets. We know that machine learning algorithms [16,17] typically operate on 2D data, but deep learning allows for the analysis of 3D data more intuitively and efficiently. 3D deep learning algorithms [18,19,20,21,22,23] can extract features such as shapes, textures, and volumes from 3D data and can be used for a wide range of applications, such as medical imaging, robotics, and virtual reality. Even though computed tomography reconstruction is a well-established technique for generating high-quality 3D images of the body, there are still several gaps in knowledge and research that require filling. Past research studies have explored aspects of CT reconstruction, but the emergence of 3D deep learning algorithms is a novel approach to enhancing image quality and efficiency.

This planned literature review aims to show the problems and opportunities in CT reconstruction that arise when 3D deep learning is used in CT reconstruction, especially when CT images are rebuilt from raw projection data into 3D data. This paper also addresses the requirement of collecting and integrating existing research, which helped the authors highlight the potential applications, challenges, and advancements of 3D deep learning in CT reconstruction. This study involved a review of the most advanced methods for 3D deep learning for CT reconstruction, its effectiveness, and its efficiency in producing high-quality 3D images. The review follows the guidelines of PRISMA [24,25,26,27] (Preferred Reporting Items for Systematic Reviews and Meta-Analyses) and also incorporates the Kitchenhand and Charters [28,29] methodology, which is specifically adapted to investigate 3D deep learning in CT reconstruction.

This SLR seeks to add to the existing literature by giving a clear picture of how 3D deep learning techniques can be used to improve and make CT image reconstruction work better. These discoveries have enhanced medical imaging research and the use of 3D deep learning algorithms for CT reconstruction. Exploring these insights may lead to improved image quality, reduced radiation exposure, and enhanced diagnostic accuracy by reconstructing CT images more precisely and effectively. This organized literature review looks into the most up-to-date and effective training and validation datasets and methods for 3D deep learning in computed tomography reconstruction. It intends to highlight the challenges faced in 3D deep learning for CT image reconstruction and to identify potential applications, improvements, and limitations. The use of 3D deep learning methods improves the quality and efficiency of CT image reconstruction by utilizing deep neural networks, resulting in exact 3D representations. By offering vital insights into their efficacy, 3D deep learning algorithms help to improve diagnostic accuracy, advance medical imaging research, and improve patient care.

## 2. Methods

### 2.1. Research Objective

The objective of this work is to examine the use of 3D deep learning in computed tomography reconstruction through an extensive survey of the literature. A systematic literature review was utilized as the research technique in this study, which is a structured and thorough method of discovering, evaluating, and analyzing published information to look into certain research concerns. We follow the well-organized recommendations provided by PRISMA [25] in this study. We also incorporate the Kitchenham and Charters [28] methodology, as well as an extension for 3D deep learning in computed tomography reconstruction investigations. In this systematic literature review, our aim is to address the following questions:

RQ1 What are the current state-of-the-art methods in 3D deep learning in computed tomography reconstruction?
RQ2 What datasets are available for training and validating 3D deep learning in computed tomography reconstruction?

This review of the literature is meant to find the best 3D deep learning methods for reconstructing computed tomography images and to find datasets that can be used to train and test these models. The purpose of the first study question is to examine the most cutting-edge techniques for 3D deep learning in CT reconstruction. The creation of deep learning algorithms is a revolutionary strategy for improving picture efficiency and quality, even though CT reconstruction is a well-established method for creating high-quality 3D images of the human body. Both scholars and practitioners may obtain insight into the most recent possibilities and breakthroughs in applications in the area by knowing the most cutting-edge approaches and techniques in 3D deep learning for CT reconstruction. Our objective was to compile current and pertinent knowledge on techniques and datasets related to 3D deep learning in computed tomography reconstruction using these eligibility criteria.

### 2.2. Data Sources and Searches

#### 2.2.1. Search String

We created a search method to find all published materials that were related to 3D deep learning in computed tomography reconstruction. By defining the population, intervention, and results in the first place, we were able to identify the keywords. As previously stated, we addressed the following study questions: “What are the state-of-the-art methods in 3D deep learning?” and “What datasets are available for 3D deep learning in computed tomography reconstruction?” Next, we determined other ways to spell the primary concepts as well as synonyms (keywords), such as “3D deep learning” and “computed tomography” for “3D reconstruction”. We checked the keywords in pertinent publications and combined the search phrases using Boolean operators like AND, OR, and NOT. (“3D deep learning” OR “deep learning”) AND (“computed tomography” OR “CT” OR “tomography reconstruction” OR “reconstruction”) AND (“3D reconstruction” OR “image reconstruction”) were the keywords we used to conduct our search as outlined in Table 1.

#### 2.2.2. Resources to Be Searched

With our current keyword list, we worked to compile all the literature that is relevant to the 3D deep learning research issues for CT reconstruction. Our selection process involved picking five databases to ensure a comprehensive search: Elsevier, Springer, MDPI, IEEE Xplore, and the Nature Publishing Group. These databases were picked because they are known for having the most representative sources for research in the fields of medicine and artificial intelligence. They are also often used in other systematic literature reviews because they have large collections of literature that are relevant to the research questions, such as journals, articles, conferences, and books. By choosing these sources, we sought to compile as many articles as we could to guarantee a thorough evaluation of the literature.

#### 2.2.3. Overview of the Search Process

We performed four basic stages to compile all the pertinent research on 3D deep learning for computed tomography reconstruction. First, we gathered primary research from the online libraries listed in the section utilizing the search phrases there. Search results were produced by the digital libraries. There were 774 publications found that were published between 2013 and 2023. In the first instance, we included every article that was cited in the original papers we picked. The second step was to remove papers that were unnecessary based on exclusion criteria. We picked the articles that satisfied the inclusion criteria from those that did not. In the second instance, we included every paper that cited the initial papers we chose. As a result of the fourth exclusion/inclusion criterion, a final selection either included or excluded the candidate. The title was the first thing we considered. The paper was skipped if it was clear that it was off-limits. As an alternative, we checked the abstract, introduction, and conclusion of each article that was thought to be possibly helpful to see if it was relevant to our study. Third, we conducted a snowballing search for any possibly misplaced papers. Given the set of sources that were eventually located using a search string and supplemented with those obtained by snowballing and manual search, for a set made up of 60 sources, 54 papers were determined to pass all phases as outlined in Table 2. After selecting the final articles for the systematic literature review, we utilized the last filtering phase, the quality assessment, to ensure that each publication provided the necessary information to answer our study questions.

### 2.3. Eligibility Criteria

#### 2.3.1. Inclusion Criteria

The reliability of 3D deep learning in CT reconstruction was considered in this review. It included both supervised and unsupervised techniques. We were looking for research that detailed 3D deep learning’s accuracy, precision, or effectiveness in computed tomography reconstruction. Additionally, we sought to include research that offered unique data, including modeling studies, observational studies, or clinical trials. We were also interested in papers that summarized the state-of-the-art methods in 3D deep learning in computed tomography reconstruction. We only took into account research that was printed in English-language journals to guarantee uniformity.

#### 2.3.2. Exclusion Criteria

Studies that primarily examined conventional image-processing methods were not included in this review, however. Additionally, we did not include studies that used imaging techniques other than deep learning. Studies that had not undergone peer review or were not available in English were also excluded from this review. Additionally, editorials, letters, or conference presentations were not taken into consideration for inclusion.

### 2.4. Quality of Evidence

In a systematic evaluation of 3D deep learning in computed tomography reconstruction, the quality of evidence is defined as the robustness and reliability of the conclusions drawn from the reviewed research. Some of the factors used to assess the quality of the evidence include the study design, bias risk, consistency of results, accuracy of estimations, and relevance and applicability of the included studies to the research topic. We evaluated the quality of the 3D deep learning in computed tomography reconstructions using inquiries:

1. Is the concept of 3D deep learning clearly defined?
2. Is the method for 3D deep learning clearly defined?
3. Are the state-of-the-art metrics explicitly reported?

When discussing the strength and reliability of the results drawn from the reviewed research on 3D deep learning in computed tomography reconstruction, the term “quality of evidence” is used. It reflects how confident we are in the veracity and applicability of the research to various contexts. Several factors are considered when judging the quality of the evidence. These include the research design, any possible biases, how consistent the results are, how accurate the estimates are, and how relevant and useful the studies included are.

For this, we had to address the above specific questions, such as the clarity of the definitions for 3D deep learning and computed tomography reconstruction, the explicit description of the method for 3D deep learning, and the reporting of cutting-edge techniques and metrics. The quality of the evidence for 3D deep learning in computed tomography reconstructions was assessed in this evaluation. The research utilized data from 18 papers on CT imaging. Additionally, we included one paper each from the HRCT and MRI modalities, along with two papers from the X-rays modality, which allowed us to explore the effectiveness of 3D deep learning techniques in medical imaging by analyzing various imaging modalities.

### 2.5. Data Extraction

The information required to address the research questions was eventually retrieved from the chosen papers. The type of 3D deep learning technique utilized for computed tomography reconstruction, the unique application domain, the dataset details, and the performance measures were all taken into account when creating the data extraction form. A further field was included to allow for the reporting of any study limitations. We started by extracting the appropriate features and evaluating the research’s shortcomings. The constraints of the investigations could be accurately and thoroughly evaluated using this cooperative method. Additionally, adding a section for restrictions made it easier to spot possible research gaps and topics for additional study. Overall, this part emphasizes the authors’ meticulous and stringent data extraction methodology, which is crucial for guaranteeing the validity and trustworthiness of a systematic literature review [30].

## 3. Background

Computed tomography (CT) is a vital medical imaging technology that revolutionizes healthcare by providing high-resolution images of internal body structures, making it an essential tool in fields like radiology, oncology, and surgery. CT imaging uses X-ray technology to scan a patient. During the CT imaging process, the patient is positioned on a motorized examination table that passes through a CT scanner. The scanner emits narrow X-ray beams, which are measured by detectors on the opposite side of the patient. The data collected are X-ray projections or profiles. CT reconstruction involves various techniques and methods to generate cross-sectional images from X-ray projection data, including the following:

### 3.1. Tomography Reconstruction

To understand 3D deep learning for computed tomography reconstruction, it is essential to understand the basic tomography reconstruction principles. Tomography is a medical imaging technique that captures cross-sectional images of the human body using X-rays or other imaging modalities. The reconstruction process transforms this data into detailed, two-dimensional (2D) or three-dimensional (3D) images representing the object’s internal structures. For turning raw projection data into useful images, it is important to use traditional tomography reconstruction methods, such as filtered back projection (FBP) and iterative reconstruction (IR) algorithms. FBP filters and back-projects data, but has limitations in sparse or irregularly sampled scenarios. Iterative reconstruction methods, on the other hand, involve an iterative optimization process to refine the image, offering advantages in handling noisy data and irregular sampling but often requiring increased computational demands. Artifacts, noise, and the requirement for a substantial amount of data can compromise the accuracy of traditional tomography reconstruction methods, leading to a reduction in image quality. This effect is particularly pronounced when employing low-dose CT or sparse-view CT, as discussed in [31].

### 3.2. Filtered Back Projection (FBP)

Filtered back projection (FBP) [32,33] plays a pivotal role in CT image reconstruction, and has revolutionized the field of medical imaging. CT imaging aims to create precise and informative images reflecting internal anatomical structures and pathological conditions. X-ray projection data is collected and then put through mathematical operations such as filtering and back projection to create cross-sectional images in two dimensions. These images are crucial for clinical interpretation, allowing physicians to visualize and analyze anatomical structures, detect abnormalities, and guide medical interventions. FBP’s [34] computational efficiency and straightforward mathematical foundation make it ideal for real-time diagnostic applications. Despite its historical importance, FBP has limitations, especially in addressing complex data corrections like scatter and beam-hardening artifacts. These limitations have spurred ongoing research and innovation in CT imaging, leading to the development of advanced reconstruction methods like iterative algorithms and deep-learning-based techniques. Filtered back projection (FBP) assumes consistent X-ray attenuation within the scanned object, which may not be consistent in some cases. It may not fully utilize raw data information, leading to potential image artifacts, and is less suitable for complex data corrections.

### 3.3. Iterative Reconstruction (IR)

Iterative reconstruction (IR) [35,36] techniques represent a revolutionary approach to reconstructing CT images, utilizing computational algorithms and iterative processes to improve image quality and reduce artifacts. IR is a paradigm shift in CT image reconstruction, focusing on a one-pass process rather than a one-pass reconstruction process. It employs an iterative approach, repeatedly refining the image based on a mathematical model that simulates the acquisition process. This process gradually converges towards a more accurate representation of the patient’s anatomy, reducing artifacts and improving image quality. IR [37] is particularly useful in scenarios with reduced radiation dose, limited projections, and prevalent noise or artifacts. It can produce high-quality images even with lower X-ray doses, mitigating health risks associated with ionizing radiation. IR’s advantages and limitations are discussed, along with its potential impact on clinical practice and its role in enhancing CT imaging. Recent advancements in computational techniques and hardware have rendered IR more accessible and effective for healthcare professionals, fundamentally reshaping the landscape of medical imaging and diagnosis.

### 3.4. Deep Learning Iterative Reconstruction (DLIR)

Deep learning iterative reconstruction (DLIR) is a revolutionary approach in medical imaging that combines deep learning with iterative reconstruction methods to produce high-quality, informative images with reduced radiation exposure. DLIR is a transformative approach that leverages deep neural networks to learn complex patterns and features from data, integrating them into the iterative reconstruction process. During each iteration, the deep learning model refines the reconstructed image, reducing artifacts and noise, and enhancing image quality. This iterative refinement process gradually converges towards a more precise representation of the patient’s anatomy, even in cases with limited data or low-dose scans. DLIR offers several advantages, including the potential to significantly reduce radiation exposure to patients without compromising image quality, making it particularly well-suited for pediatric imaging. It also excels in scenarios with challenging data, such as metal artifacts or limited projections, where traditional reconstruction methods may fall short. DLIR’s fundamental principles and mechanics will be explored, along with its advantages and limitations, its potential impact on clinical practice and healthcare, and its ongoing evolution. How DLIR is becoming increasingly accessible and efficient due to advancements in computational technology, reshaping the landscape of medical imaging and diagnosis, will also be examined.

### 3.5. Deep Learning Reconstruction (DLR)

Deep learning reconstruction (DLR) [38] is a revolutionary approach in medical imaging that improves the quality and speed of image reconstruction. It uses deep neural networks, a subset of artificial intelligence, to enhance the reconstruction of medical images, such as those obtained from CT scans or MRIs. Traditional methods, like filtered back projection (FBP) and iterative reconstruction (IR), have limitations, especially when dealing with noisy data or rapid reconstruction. DLR introduces a transformative approach by integrating deep neural networks into the image reconstruction process, which learns complex patterns and features from the acquired data. DLR can adapt and optimize the reconstruction process based on the specific data it processes, enhancing image quality by reducing artifacts, noise, and imperfections. This is particularly useful in real-time or near-real-time image-generation scenarios, such as interventional radiology or emergency medical situations. DLR’s [39] fundamental principles and mechanics are explored, along with its potential impact on clinical practice and healthcare. Advancements in computational technology have made DLR more accessible and efficient, ultimately reshaping the landscape of medical imaging and diagnosis.

## 4. Results

### 4.1. Search and Study Selection

We initiated our search by exploring five databases to ensure a systematic literature review, resulting in the identification of 774 documents. A total of 66 duplicate entries were eliminated, leaving 696 unique records for further analysis. We carefully examined the titles of the 696 records during the screening phase and eliminated 442 items that did not fit our predetermined inclusion criteria. Subsequently, we thoroughly evaluated the abstracts of the remaining 254 records. Among these, 113 papers were disqualified for a variety of reasons, including not fitting the qualifying requirements or being deficient in pertinent data.

**Figure 2:**
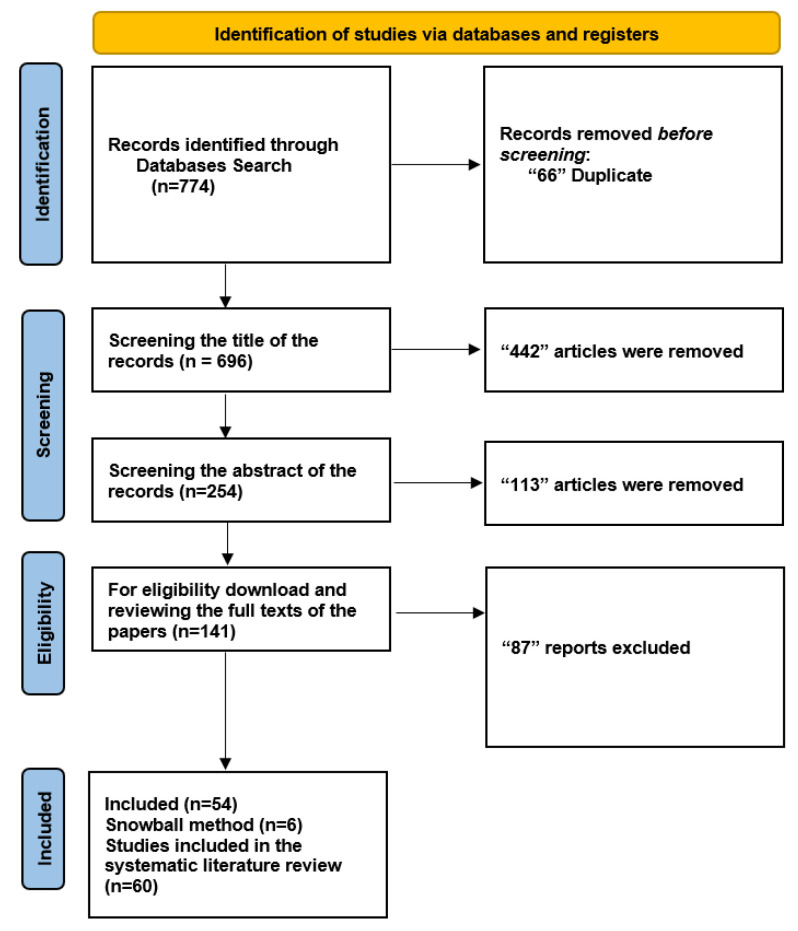
Flowchart: The study involved a comprehensive search of over 60,000 abstracts and ultimately selected 10 studies for meta-analysis and 2 studies for qualitative synthesis, with a focus on the application of 3D deep learning in computed tomography reconstruction, and presents the findings in tables summarizing diagnostic accuracy metrics for imaging and several specialties.

We collected and thoroughly read the complete texts of 141 articles to make sure they were eligible. Based on our predefined criteria, 87 reports were eliminated from this group. As a result, the 54 papers that met the inclusion requirements were incorporated into our systematic literature review, providing the information necessary for sensitivity and specificity assessments. We used a snowball strategy [40] in addition to our original search to find more connected publications. Utilizing this method, we identified an additional six publications, increasing the total number of included studies to 60. A thorough overview of the results is provided in Figure 2. The study also explores the relationship between CT image reconstruction and diagnostic accuracy, highlighting the impact of 3D deep learning techniques on image quality and diagnostic performance.

### 4.2. RQ1 What Are the Current State-of-the-Art Methods in 3D Deep Learning in Computed Tomography Reconstruction?

Understanding the current state-of-the-art methodologies in 3D deep learning in computed tomography reconstruction is essential for researchers and practitioners alike. The primary aim of this systematic literature review is to provide a review of the current state-of-the-art methods in 3D deep learning in computed tomography reconstruction. The review was carried out by searching through relevant journals and choosing studies based on predefined inclusion criteria. This ensured that all of the most recent developments were covered.

The presented Table 3 provides an overview of 3D deep learning methods for computed tomography reconstruction, encompassing various methodologies and their outcomes in medical image analysis, serving as a valuable resource for researchers, clinicians, and anyone interested in the intersection of deep learning and medical imaging. It showcases the diverse range of state-of-the-art methods and their potential to impact various medical applications, while offering a global perspective on their development and adoption.

Table 3 presents a comprehensive overview of recent advancements in medical imaging, particularly in the field of computed tomography (CT) reconstruction. Researchers worldwide have contributed to this field, showcasing diverse methodologies and their respective population performance metrics. Some notable studies include those by Setio, Li, Meng, Wang, Gruetzema, Gu, Yu, Ren, and Xuhua. These studies have achieved impressive accuracy rates, sensitivity, and specificity in CT detection, improved reconstruction metrics, and enhanced segmentation accuracy. In other research, CNN has been used in CT scans to identify pneumothorax with high sensitivity and specificity and to improve reconstruction metrics. Annarumma achieved 100% sensitivity and 96% specificity for lung nodule classification, while Lee H in the UK focused on CNN for radiograph triaging. The diversification extends beyond CT, with studies exploring DNN-MPRAGE in MRI and comparing DLIR-H in CT. These studies contribute to the evolving landscape of medical imaging, pushing the boundaries in accuracy, speed, and radiation reduction.

#### 4.2.1. Low-Dose CT Reconstruction

The popularity of low-dose CT imaging has grown significantly owing to its effectiveness in reducing radiation exposure. Studies by Li, Meng [42] and Uthoff [49] illustrate the effectiveness of 3D CNNs in achieving high sensitivity and specificity, ensuring accurate diagnostic outcomes. These advancements not only improve patient safety but also demonstrate deep learning’s potential to maintain diagnostic accuracy even with reduced radiation doses.

#### 4.2.2. Sparse-View CT Reconstruction

Researchers such as Park, Sungeun [55] and Higaki, Toru [38] have explored novel techniques, including deep learning algorithms and cycleGAN, to address challenges in sparse-view CT reconstruction. These methods effectively reconstruct images from limited data views, providing a potential solution to the problem.

#### 4.2.3. Country-Based Analysis

**Figure 3:**
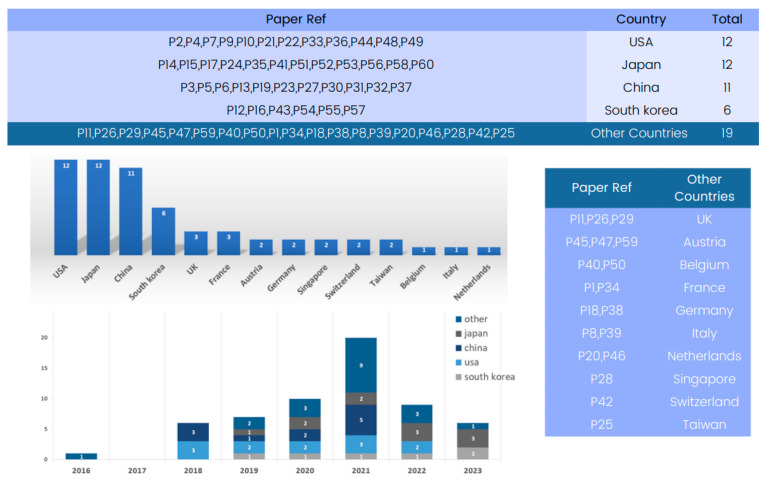
Global contribution to 3D deep learning research in computed tomography reconstruction over the years.

Figure 3 provides a country-based analysis of the state-of-the-art 3D deep learning methods applied to computed tomography reconstruction. A review of the “Country” column reveals a global view of innovation in this field.

Several countries have emerged as significant contributors to the advancement of 3D deep learning in computed tomography reconstruction. Notably, the United States and Japan have multiple entries, reflecting their foundational role in developing deep learning methods for CT reconstruction, particularly in lung nodule classification and pneumothorax detection. China and Republic of Korea also appear strongly, with numerous submissions indicating significant contributions in areas such as CT segmentation and image denoising.

#### 4.2.4. Database-Driven

**Figure 4:**
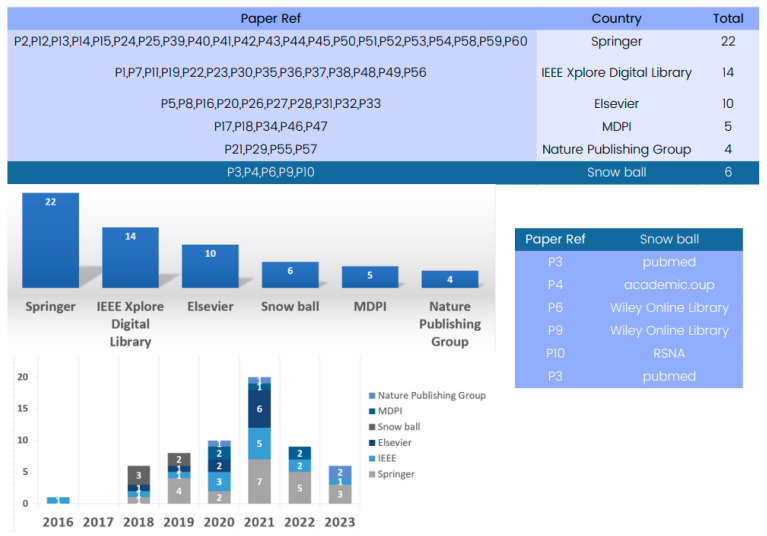
Evolution of databases utilized in 3D deep learning for computed tomography reconstruction over the years.

The state-of-the-art methods for 3D deep learning in computed tomography (CT) reconstruction have significantly improved patient care and diagnosis, as evidenced by the comprehensive database analysis presented in Figure 4.

The database analysis reveals numerous research articles on 3D deep learning for CT reconstruction, with Springer being a key platform with 22 papers for showcasing advancements. IEEE and Elsevier have also contributed significantly to the field, with numerous articles highlighting the impressive performance of deep learning models in CT reconstruction.

The database analysis emphasizes the crucial role played by prominent publishers in advancing research on 3D deep learning methods in CT reconstruction, thereby shaping the future landscape of medical imaging.

#### 4.2.5. Methodology Review

**Figure 5:**
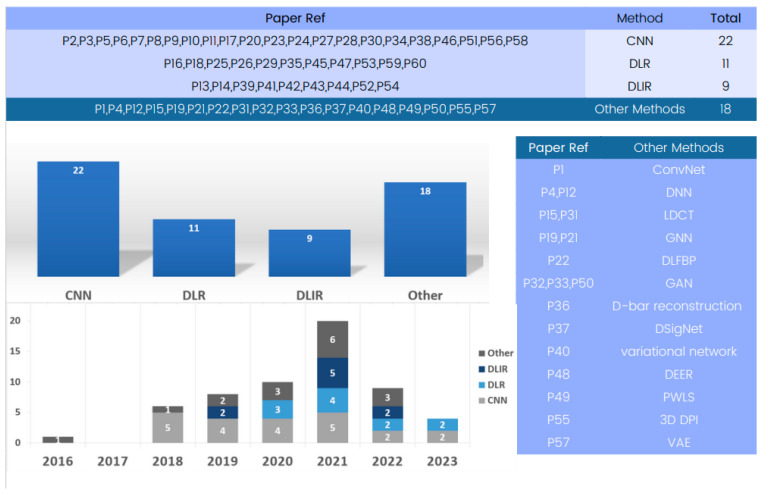
Evolution of 3D deep learning methods in computed tomography reconstruction over the years.

The included papers’ analyses showed a variety of models and methods used in 3D deep learning in computed tomography reconstruction. An examination of Figure 5 reveals methodologies employed in the field of 3D deep learning in CT reconstruction. Among these methodologies, convolutional neural networks (CNNs) emerge as an important method for research and are also prominently featured in numerous entries.

We include 3D CNNs in the category of CNNs, which are designed to operate on volumetric data and offer improved spatial awareness and accuracy compared to their 2D counterparts. Their three-dimensional convolutional layers allow for more in-depth understanding of anatomical components, making them appropriate for tasks such as segmentation. CNNs are widely accepted in medical imaging due to their consistent usage and effectiveness. They consistently achieve high sensitivity, specificity, and accuracy in tasks like lung nodule classification and pneumothorax detection, demonstrating their enduring relevance and relevance.

Another noteworthy methodology is deep learning reconstruction (DLR) [94], a widely used method that significantly improves image quality and reduces radiation dose. It offers enhanced performance and shorter reconstruction times compared to traditional methods, like filtered back projection (FBP).

Additionally, Figure 5 presents various methods, like deep learning iterative reconstruction (DLIR), variational autoencoders (VAEs), and generative adversarial networks (GANs) [95,96], to tackle medical imaging challenges, enhancing the accuracy, efficiency, and quality of medical image analysis, showcasing the field’s dynamic and innovative nature.

Deep learning reconstruction (DLR) serves as a versatile approach that integrates various methods, including convolutional neural networks (CNNs), generative adversarial networks (GANs), variational autoencoders (VAEs), and recurrent neural networks (RNNs). Many of the reviewed papers utilize DLR as a comprehensive framework for 3D deep learning in computed tomography reconstruction, unifying multiple methods to enhance image quality, reduce radiation dose, and improve overall performance. It is worth noting that some papers mention DLR without specifying individual methods.

#### 4.2.6. Convolutional Neural Networks (CNN)

For a wide range of computer vision applications [97], convolutional neural networks (CNNs) [98,99,100] have become a dominant deep learning model. Given their efficiency in learning hierarchical features from CT images, CNNs play a crucial role in computed tomography reconstruction. CNNs [101] analyze the input data in the context of computed tomography reconstruction by applying several trainable filters that convolve across the input’s structural dimensions. Local patterns and characteristics may be extracted from the computed tomography data using this convolution procedure [102,103]. CNNs may learn representations that are especially suited to added tomography reconstruction tasks by stacking many convolutional layers, which allows them to capture increasingly complicated and abstract aspects from the input data. The hierarchical structure of CNNs enables them to learn features at various abstraction levels. Higher layers of the network learn more sophisticated and meaningful representations, whereas lower levels tend to capture simpler elements, like edges and textures. Due to their capacity to extract hierarchical features, CNNs are highly suited for computed tomography reconstruction [104,105,106] because they can identify important structures and patterns in volumetric CT images.

In this research article on 3D deep learning in computed tomography reconstruction, the use of CNNs is constantly emphasized as a critical strategy. These studies demonstrate how CNNs are widely used for computed tomography reconstruction tasks in a variety of variants and topologies. The extensive use of CNNs underscores their effectiveness in enhancing reconstruction accuracy [107] by leveraging learned characteristics from CT images.

These models may learn to recognize common patterns and structures present in the data by training CNNs on massive datasets of CT images. By assuming missing information from the input scans, CNNs can produce high-quality reconstructions [108,109,110,111]. In comparison to conventional techniques, CNN-based computed tomography reconstruction models have demonstrated significant advancements [112], yielding reconstructions that exhibit higher precision and aesthetic appeal. Furthermore, enhancements in regularization methods can prove beneficial for the application of CNNs in computed tomography reconstruction. Computed tomography reconstruction has been made more effective and efficient by using deep learning regularization (DLR), which typically refers to the use of regularization in machine learning or neural network models to prevent overfitting and enhance model generalization [113,114] using techniques such as the efficient dense learning framework (EDLF). These regularization techniques make use of techniques to improve deep learning models, producing better reconstruction outcomes. Their incorporation also enhances the effectiveness of regularization-based computed tomography reconstruction models. The use of CNNs in computed tomography reconstruction has a lot of potential to advance the science and to advance medical imaging applications.

#### 4.2.7. 3D Convolutional Neural Networks (3DCNN)

Operating on volumetric data like 3D computed tomography scans [115], 3D convolutional neural networks (3D CNNs) [116] have become a potent extension of conventional CNNs. 3D CNNs expand convolutional procedures to capture spatial relationships and complicated patterns throughout the entire volume, in contrast to typical CNNs that analyze 2D pictures individually. A significant advantage of 3D CNNs in computed tomography reconstruction lies in extending convolutional processes to the third dimension. 3D CNNs [117,118,119] can efficiently capture the spatial interdependence and connections that exist within computed tomography scans by taking into account the entire 3D environment of the volumetric data. The precision and robustness of reconstruction approaches are increased as a result of 3D CNNs’ enhanced ability to grasp the complex interactions between various areas and structures.

Research papers on 3D deep learning in computed tomography reconstruction frequently examine the usage of 3D CNNs as a cutting-edge model architecture. These studies show the benefits of employing 3D CNNs for reconstruction as well as their ability to recognize complex patterns and features in CT images. 3D CNNs improve the accuracy of reconstruction by using the volumetric part of the data to include contextual connections and spatial information. Both of these are needed for correct reconstruction. An ongoing project is to apply cutting-edge architectural designs to improve reconstruction results using 3D CNNs in computed tomography reconstruction. By augmenting CNNs’ ability to analyze volumetric data, researchers have overcome the limitations of traditional 2D-based methods. Computed tomography reconstruction using 3D CNNs can capture the intricate anatomical details visible in volumetric CT images. Due to the data’s multidimensionality, 3D CNNs can recognize and comprehend the connections between neighboring slices, which improves the quality of the complete volume reconstruction. This capacity is especially important when the structures of interest span several slices or have complex spatial arrangements.

3D CNNs gain from improvements in regularization methods in addition to their capacity to capture spatial relationships. To further improve the accuracy and robustness of reconstruction approaches, deep neural networks (DNNs) with more complicated architectural designs have been used.

The extensive adoption of 3D CNNs in academic publications attests to their effectiveness in enhancing reconstruction accuracy and their role in advancing the field of computed tomography reconstruction. By using the whole 3D context of computed tomography scans, 3D CNNs offer insights about the intricate structures seen in medical imaging, enabling higher-quality reconstructions and more accurate medical diagnosis and treatment.

### 4.3. RQ2 What Datasets Are Available for Training and Validating 3D Deep Learning in Computed Tomography Reconstruction?

Understanding the available datasets for training and validating 3D deep learning in computed tomography reconstruction is essential for researchers and practitioners alike. The primary aim of this systematic literature review is to provide a review of available datasets for training and validation in 3D deep learning in computed tomography reconstruction.

The review was conducted through a systematic search of relevant journals and selected studies based on predefined inclusion criteria, ensuring comprehensive coverage of recent developments.

The presented Table 4 provides an overview of 3D deep learning methods for computed tomography reconstruction, encompassing various methodologies and their outcomes in CT images, serving as a valuable resource for researchers, clinicians, and anyone interested in the intersection of 3D deep learning and medical imaging. It showcases the diverse range of available datasets and their potential to impact various CT reconstructions.

#### 4.3.1. Country-Based Analysis

**Figure 6:**
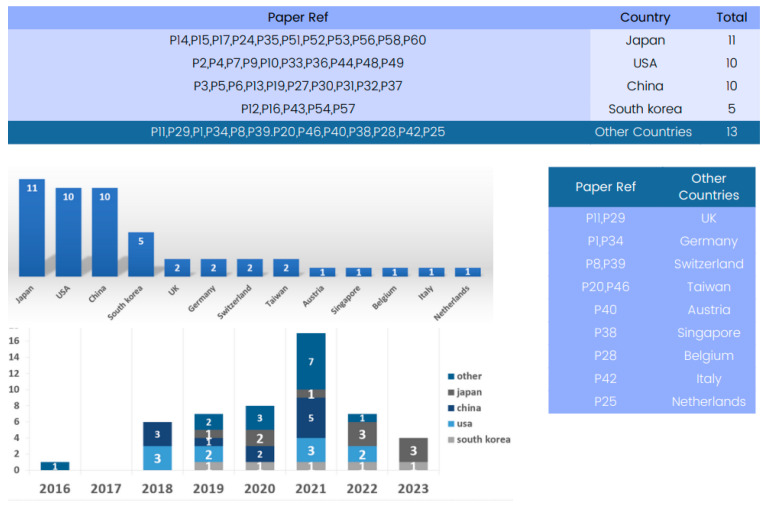
Global contribution available datasets for training and validation in 3D deep learning research in computed tomography reconstruction over the years.

Figure 6 provides a country-based analysis of available datasets for training and validation in 3D deep learning methods applied to computed tomography reconstruction.

A complete review of the “Country” column reveals a global view of innovation in this field. Several countries stand out as significant contributors to the advancement of 3D deep learning in computed tomography reconstruction. Based on the review, Japan, the United States, China, and Republic of Korea have made substantial contributions to this field, providing datasets from various medical imaging modalities. These datasets vary in size, content, and application, catering to different aspects of CT reconstruction research. Researchers worldwide have the opportunity to access and utilize these datasets to advance 3D deep learning in CT reconstruction.

#### 4.3.2. Database-Driven Evaluation

**Figure 7:**
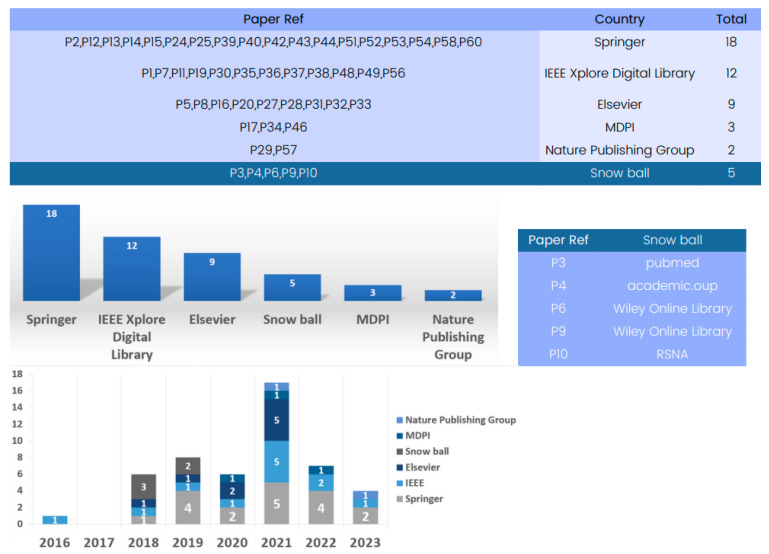
Evolution of databases utilized for available datasets for training and validation in 3D deep learning for computed tomography reconstruction over the years.

Available datasets for training and validating 3D deep learning in computed tomography reconstruction have significantly improved patient care and diagnosis, as evidenced by the comprehensive database analysis presented in Figure 7.

These databases are sourced from various reputable publishers, such as IEEE, Springer, and Elsevier, encompassing datasets primarily focused on CT imaging. Springer serves as a key platform with 18 papers showcasing advancements. Additionally, IEEE and Elsevier have significantly contributed to the field, featuring numerous articles that underscore the impressive performance of available datasets for training and validating 3D deep learning in computed tomography reconstruction.

#### 4.3.3. Dataset Review

**Figure 8:**
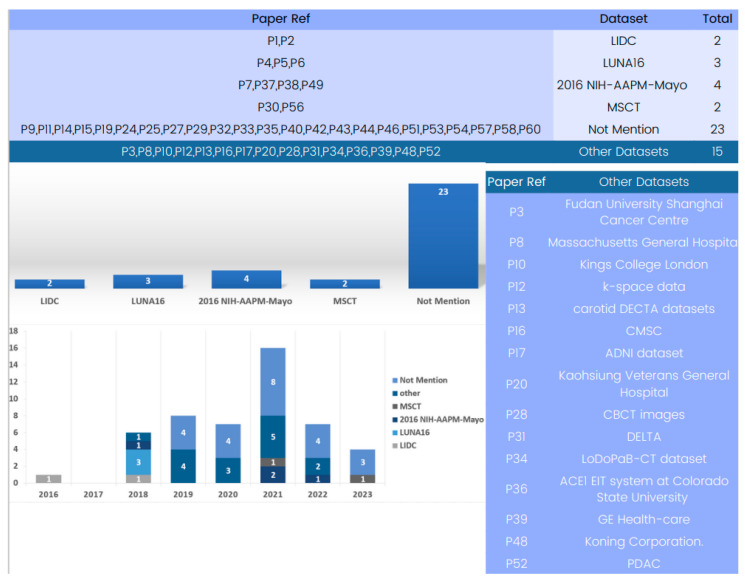
Evolution of 3D deep learning datasets in computed tomography reconstruction over the years.

Several datasets have been employed in 3D deep learning for computed tomography (CT) reconstruction, as indicated by the analysis of the included papers. An examination of Figure 8 reveals the available datasets for training and validating 3D deep learning in computed tomography reconstruction. Some studies utilized the LIDC-IDRI dataset [122], which offers an extensive collection of lung nodule CT scans, providing crucial data for the development and evaluation of CT reconstruction models. However, this dataset has not been used in research after 2017. Similarly, LUNA16 [123], employed in three different studies, is a frequently cited dataset but was primarily used in 2018 for testing nodule detection algorithms and has seen limited usage since then.

In the past few years, the 2016 NIH-AAPM-Mayo and MSCT datasets have become advanced and useful tools for 3D deep learning in CT reconstruction. Notably, several papers use only patient datasets which do not explicitly define the dataset source due to the use of patient data.

The study of datasets used in computed tomography reconstruction with 3D deep learning shows how varied and specific the data used in this field is. For training and evaluating computed tomography reconstruction models in a variety of settings, from lung nodule identification to HRCT and X-ray-based reconstruction, datasets like 2016 NIH-AAPM-Mayo and MSCT are accessible and offer helpful resources. Researchers should carefully consider the characteristics, size, and clinical value of the datasets when selecting the most suitable data for their specific applications. By using a range of datasets, researchers may enhance the robustness, generalizability, and clinical usefulness of 3D deep learning models in computed tomography reconstruction.

#### 4.3.4. Lung Nodule Analysis 2016 (LUNA16)

The study and development of 3D deep learning for computed tomography reconstruction has greatly benefited from the dataset LUNA1651 [124,125] (Lung Nodule Analysis 2016). Since LUNA16 was developed primarily for evaluating nodule detection algorithms, it has gained recognition as a useful tool for creating and validating computed tomography reconstruction models. One of LUNA16’s key benefits is the vast array of lung nodule pictures it offers. A total of 1186 CT pictures make up the collection, which offers a wide range of examples and annotations pertaining to lung nodules. Due to this variability, deep learning models may be created and tested on a large range of instances, accurately representing the heterogeneity and complexity of lung nodules seen in actual computed tomography scans.

Due to the significance of LUNA16 as a benchmark dataset, researchers frequently use it. The performance and generalizability of deep learning models in the context of lung nodule recognition and CT reconstruction are evaluated using this standardized assessment approach. Researchers may evaluate various strategies, algorithms, and methods using LUNA16, making it easier to pinpoint the benefits and drawbacks of computed tomography reconstruction models as well as potential areas for development.

For researchers, the annotations offered by LUNA16 are of great use. These annotations contain ground truth labels and nodule delineations, providing deep learning algorithms with trustworthy benchmarks for training and assessment. The presence of such annotations greatly improves the efficacy and accuracy of the created models.

Many research papers talk about LUNA16 and how important it is for testing and measuring how accurate and useful computed tomography reconstruction models are. The dataset has evolved into a benchmark for comparing and assessing the effectiveness of various algorithms, giving academics a uniform yardstick for judging their strategies. The frequent use of LUNA16 in academic works emphasizes how important it is as a trustworthy and extensively used dataset in the area.

Furthermore, improvements in lung nodule identification and computed tomography reconstruction have been made because of the use of LUNA16. Researchers have improved the detection and reconstruction of lung nodules by using the dataset to build novel deep-learning architectures, feature extraction methods, and post-processing approaches. Researchers can improve the precision and effectiveness of computed tomography reconstruction and, hence, aid in the early identification and diagnosis of lung illnesses by training and evaluating their models using LUNA16.

The fact that LUNA16 is used so often in research papers shows how useful this framework is as a standard evaluation tool and a reliable source for checking the accuracy and usefulness of CT reconstruction models.

#### 4.3.5. Lung Image Database Consortium–Image Database Resource Initiative (LIDC-IDRI)

The study of 3D deep learning in computed tomography reconstruction has greatly benefited from the dataset LIDC-IDRI [126] (Lung Image Database Consortium–Image Database Resource Initiative). It is commonly used and extensively cited in research works in this field. An important resource for the creation and assessment of computed tomography reconstruction models, LIDC-IDRI has a comprehensive collection of about 1186 CT images.

The extensive and varied collection of lung nodule scans that LIDC-IDRI includes is one of its main advantages. Researchers may use real-world computed tomography scans to train and test deep learning algorithms since the dataset provides a wide variety of examples and annotations pertaining to lung nodules.

Deep learning methods for computed tomography reconstruction have advanced and have been improved greatly as a result of the use of LIDC-IDRI. The use of this dataset has allowed researchers to refine their models and explore new avenues for increasing the precision, robustness, and generalizability of computed tomography reconstruction methods. Due to the availability of LIDC-IDRI, it is now easier to evaluate the efficiency and performance of various algorithms, allowing academics to compare and verify their models against industry standards.

The prominence of LIDC-IDRI in the area is demonstrated by the volume of references to and citations of its work in various research articles. Researchers frequently use LIDC-IDRI to confirm the effectiveness of their suggested methods, highlighting the significance and dependability of this dataset.

Radiology as a whole and computed tomography reconstruction methods have advanced as a result of the use of LIDC-IDRI in research. Researchers have utilized the LIDC-IDRI dataset to develop deep learning architectures, feature extraction strategies, and post-processing approaches for CT scan reconstruction. This has led to more accurate and effective models, and evaluation of their effectiveness, robustness, and generalizability. The dataset’s extensive use in academic publications highlights its crucial role in advancing 3D deep learning in computed tomography reconstruction.

#### 4.3.6. 2016 NIH-AAPM-Mayo

The 2016 NIH-AAPM-Mayo [127] dataset is a collection of CT images used in the “2016 Low-Dose CT Grand Challenge [128]”, sponsored by the National Institutes of Health, the American Association of Physicists in Medicine, and the Mayo Clinic. The dataset focused on CT reconstruction and denoising techniques and was made publicly available to the scientific community. Researchers worldwide have used this dataset to improve CT image reconstruction and noise reduction techniques, facilitating further research and development in the field of medical imaging.

The 2016 NIH-AAPM-Mayo dataset [129], used in research papers P07, P37, P38, and P49, has significantly aided in the development of deep learning techniques for CT reconstruction. The dataset, which comprises 500 CT images with 1493 pixels in the view direction and 720 views, has been instrumental in improving image quality and enhancing CT reconstruction methods. In P37 and P38, the dataset was used to train deep learning models, allowing for the exploration of advanced CT reconstruction techniques.

The dataset’s large number of slices and epochs has facilitated robust model training, leading to improved CT reconstruction outcomes. In P49, the dataset was used to obtain LDCT and SDCT images with a size of 512 × 512. This made it possible to test different CT image reconstruction methods, especially those used in low-dose CT imaging. This allowed researchers to assess and compare the performance of other reconstruction methods, leading to improvements in image quality and dose-reduction strategies. The 2016 NIH-AAPM-Mayo dataset has proven to be a valuable resource for researchers, resulting in advancements in CT reconstruction techniques and enhanced deep learning approaches in CT imaging.

#### 4.3.7. MSCT

Multi-slice computed tomography (MSCT) [130] is a non-invasive medical imaging technique that uses X-ray beams and liquid dye to create 3D images of the heart and blood vessels. Unlike traditional coronary angiography, which injects dye into a vein, MSCT injects the dye into a superficial vein. The dye travels through the bloodstream to the coronary arteries, where a CT scanner scans it. The procedure is quick, safe, and requires minimal radiation exposure, making it a safer option for cardiac health assessment.

MSCT datasets [131] have significantly improved CT reconstruction techniques, resulting in sharper and more detailed images as reported in research papers P30 and P56. They have also enhanced the capabilities of deep learning models for CT image reconstruction, and their widespread application across various clinical scenarios highlights their significance in medical imaging.

## 5. Discussion

In this systematic literature review, we explored and analyzed contemporary state-of-the-art methods for 3D deep learning in CT reconstruction. In addition, we also worked on the availability of datasets to train and validate these methods. By conducting this review, the authors aimed to identify the techniques and approaches used in CT reconstruction and to assess the datasets available for training and validating these models. This paper chiefly focuses on the challenges associated with conventional computed tomography image reconstruction techniques, such as filtered back-projection (FBP), which often results in artifacts and noise that affect the image quality and diagnostic accuracy. The study of deep learning algorithms offered an auspicious solution for the improvement of the quality and competence of CT image reconstruction.

The authors recognized the ability of 3D deep learning to produce more accurate and detailed 3D pictures, hence boosting the diagnostic capabilities of CT scans. The purpose of this study was to enhance the knowledge and uptake of these novel techniques in the field of medical imaging by examining the state of research and developments in 3D deep learning for CT reconstruction.

This systematic literature review has accomplished all its goals. The researchers conducted a comprehensive search on five major databases, identified 60 relevant research articles, and followed the Preferred Reporting Items for Systematic Review and Meta-Analysis (PRISMA) statement to ensure the quality and rigor of the review process. Convolutional neural networks and 3D convolutional neural networks were shown to be the most utilized 3D deep learning approaches for CT reconstruction through this investigation.

In addition, two significant datasets, the 2016 NIH-AAPM-Mayo and MSCT, were recognized as acceptable sources for training and evaluating 3D deep learning systems in CT reconstruction. The primary conclusions of the systematic analysis of the literature underlined the widespread use of CNNs and 3D CNNs in the field of deep-learning-based CT image reconstruction. These cutting-edge algorithms have the potential to increase CT image reconstruction’s efficacy and efficiency, resulting in better picture quality and perhaps even less radiation exposure for patients.

Consideration of the 2016 NIH-AAPM-Mayo and MSCT datasets revealed important information on the availability of pertinent data for developing and analyzing deep learning models in the context of CT reconstruction. The study has important ramifications for diagnostic radiology and the field of medical imaging. The research illustrates the use of 3D deep learning in CT reconstruction, highlighting the potential to improve patient outcomes and diagnostic precision. Adoption of these deep learning techniques has the potential to transform CT reconstruction, making it more accurate and effective, which would be advantageous to patients and healthcare systems alike.

The article concludes that 3D deep learning, especially when using CNNs and 3D CNNs, has enhanced CT reconstruction. These methods may be able to overcome the limitations of traditional imaging techniques, improving image quality, improving diagnostic accuracy, and possibly reducing radiation exposure. In addition, the identification of the datasets that the 2016 NIH-AAPM-Mayo and MSCT approaches provide offers valuable resources for researchers and practitioners interested in developing and validating deep learning models in CT reconstruction.

## 6. Limitaion of the Literature

There are several limitations to the literature on 3D deep learning in computed tomography reconstruction. Firstly, there is a lack of variety in the test datasets, with an emphasis on particular datasets, which cannot accurately reflect the larger difficulties in computed tomography reconstruction across different bodily systems and disorders. Small sample sizes may limit the generalizability of the results in some research, necessitating larger and more varied datasets for validation. Furthermore, the ability to compare the effectiveness of 3D deep learning models across various populations and imaging techniques is hampered by the lack of external validation. Additionally, it is not easy to fully comprehend the relative performance of multiple methodologies or procedures without comparative analysis. Furthermore, there has been little investigation into imaging techniques outside of computed tomography. Finally, there is a need to close the gap between research results and clinical application in the real world, taking into account elements like clinical workflow integration and validation in sizable clinical trials. By overcoming these restrictions, 3D deep learning in computed tomography reconstruction will become more reliable, generalizable, and clinically applicable, opening the door for its effective use in clinical settings.

## 7. Conclusions

The systematic literature review explores the current state-of-the-art methods and datasets for training and validation in 3D deep learning for computed tomography (CT) reconstruction. To achieve our goals, we searched major databases and identified relevant research papers. The study has revealed that convolutional neural networks (CNNs), 3D convolutional neural networks (3D CNNs), and deep learning reconstruction (DLR) are the most frequently utilized approaches in this specific field. Moreover, the paper also focuses on the accessibility of datasets for training and validating 3D deep learning models in CT reconstruction. The 2016 NIH-AAPM-Mayo and MSCT datasets were recognized as valuable sources suitable for the purpose of research, highlighting the transformative impact of adopting 3D deep learning techniques in medical imaging, offering the promise of improved patient outcomes and diagnostic precision. By providing a perspicuous insight into both research questions, the paper offers a comprehensive understanding of the current advancements and resources in 3D deep learning in CT reconstruction.

3D deep learning in CT reconstruction faces challenges due to limited test datasets, small sample sizes, and limited external validation. Comparative analysis and real-world application are needed to improve reliability, generalizability, and clinical applicability. Future studies should focus on real-time reconstruction techniques, integrating imaging modalities, and enhancing interpretability. Collaborations between scientists, doctors, and data scientists can create standardized datasets, assessment measures, and benchmarking frameworks for improved precision and effectiveness in medical imaging applications.

## Figures and Tables

**Table 1:** Database search strings.

Database	Search String
Elsevier	3D + deep learning + computed tomography + reconstruction
MDPI	3D deep learning AND computed tomography AND reconstruction
Nature	3D deep learning AND computed tomography AND reconstruction
IEEE	(“All Metadata”: deep learning) AND (“All Metadata”: computed tomography) AND (“All Metadata”: reconstruction)
Springer	3D deep learning + computed tomography + reconstruction

**Table 2:** Search results and information sources.

Resource Name	Total Results Found	Final Selection
IEEE Xplore Digital Library	30	14
Springer	31	22
Elsevier	27	10
MDPI	25	5
Nature Publishing Group	22	4
Snowball	6	6
Total	141	60

**Table 3:** Overview of state-of-the-art 3D deep learning methods in computed tomography reconstruction.

Author	Method	Population	Performance	Country	Year	Database	Ref.
Setio [41]	ConvNet	CT	85.4% and 90.1% at 1 and 4 false positives per scan	Germany	2016	IEEE	P01
Li, Meng [42]	3D ECNN	CT	PSNR = 29.3087, SSIM = 0.8529	USA	2018	Springer	P02
Wang [43]	3D CNN	HRCT	84.0% accuracy, 88.5% sensitivity, 80.1% specificity, AUC 89.2%	China	2018	pubmed	P03
Gruetzema [44]	DNN	CT	89.29% detection rate, 94.21% sensitivity, 1.789 false positives/scan	USA	2018	https://academic.oup.com/	P04
Gu, Yu [45]	3D CNN	CT	87.94% sensitivity, 92.93% at 4 FPs/scan	China	2018	Elsevier	P05
Ren, Xuhua [46]	3D CNN	CT	Higher segmentation accuracy (DC: 0.58–0.71, 95HD: 2.23–2.81 mm)	China	2018	Wiley Online Library	P06
Gupta H [47]	CNN	CT	High sensitivity (100%), specificity (82.5%) for pneumothorax detection	USA	2018	IEEE	P07
Li, Xiang [48]	CNN	CT	Slightly increased reconstruction (27.02 dB) with reduced training time (50%)	Switzerland	2019	Elsevier	P08
Uthoff [49]	CNN	CT	High sensitivity (100%) and specificity (82.5%) for pneumothorax detection	USA	2019	Wiley Online Library	P09
Annarumma [50]	CNN	CT	100% sensitivity, 96% specificity for lung nodule classification	USA	2019	RSNA	P10
Lee H [51]	CNN	CT	Sensitivity 71%, specificity 95% for normal radiographs triaging	UK	2019	IEEE	P11
Jung, Woojin [52]	DNN-MPRAGE	MRI	DNN-MPRAGE reduced acquisition time by 38%	Republic of Korea	2019	Springer	P12
Jiang, Chenyu [53]	DLIR-H	CT	DLIR-H significantly improved image quality, noise, and texture compared to ASIR-V. DLIR-L and DLIR-M showed comparable denoising.	China	2019	Springer	P13
Sato, Mineka [54]	DLIR	CT	DLIR significantly reduced image noise, improved CNR, vessel conspicuity, overall image quality	Japan	2019	Springer	P14
Park, Sungeun [55]	LDCT	CT	LDCT using DLD with 67% dose reduction showed non-inferior overall image quality and lesion detectability compared to SDCT.		2019	Springer	P15
Higaki, Toru [38]	DLR	CT	33.3% dose non-inferior to MBIR at 100% for liver lesion detection using LDCT	Republic of Korea	2020	Elsevier	P16
Singh, Satya P [20]	3D CNN	MRI	Discussing the challenges and future trends of 3D CNNs and deep learning models in medical imaging.	Japan	2020	MDPI	P17
Lenfant, Marc [25]	DLR	CT	DLR significantly improved image quality and reduced radiation dose compared to hybrid-IR in CTPA examination	Singapore	2020	MDPI	P18
Zhang J [56]	EDLF-CGAN algorithm	CT	Compared to traditional algorithms, EDLF-CGAN showed superior SR reconstruction effects	China	2020	IEEE	P19
Liang, C-H [57]	CNN	X-rays	76.6% sensitivity, 88.68% specificity	Taiwan	2020	Elsevier	P20
Wang, Ge [58]	cycleGAN	CT	Deep learning algorithms for tomographic imaging are data-driven and must continually evolve to accommodate new data sources.		2020	Nature Publishing Group UK London	P21
Fu J [59]	DLFBP	DPC-CT	The proposed framework achieves improved imaging quality, faster processing		2020	IEEE	P22
Jiao F [60]	CNN(iBP-Net)	CT	The experimental validation demonstrates the efficacy of iBP-Net in CT reconstruction.	China	2020	IEEE	P23
Ichikawa [61]	CNN-based	CT	The proposed deep-learning method showed clinically acceptable accuracy for estimating body weights from CT scout images.	Japan	2020	Springer	P24
Oostveen [62]	DLR	CT	DLR showed superior image quality, and shorter reconstruction times (27 s DLR, 44 s Hybrid-IR, 176 s MBIR).	Netherlands	2020	Springer	P25
McLeavy [39]	DLR	CT	DLR uses AI and supercomputer technology for high image quality, low radiation dose	UK	2021	Elsevier	P26
Zeng [63]	CNN	CT	LDCTDL showed 73.5% sensitivity and 82.4% specificity.	China	2021	Elsevier	P27
Verhelst [64]	CNN	CT	AI and RAI scored an IoU of 94.6% and 94.4%, respectively.	Belgium	2021	Elsevier	P28
Aggarwal [65]	DL algorithm	CT	DL algorithms demonstrated high diagnostic accuracy in identifying various diseases.	UK	2021	Nature Publishing Group UK London	P29
Han XF [66]	CNN	CT	The 2.5D method outperformed other 2D, 2.5D, and 3D methods in drowning diagnosis.	China	2021	IEEE	P30
Zeng [63]	LDCTDL	CT	LDCTDL showed lower noise, higher SNR and CNR compared to SDCTHIR and LDCTHIR, maintaining image quality.	China	2021	Elsevier	P31
Jiang, Hao [67]	CycleGAN	CT	Deep learning models show excellent accuracy, precision, recall, and F1 score in COVID-19 classification using synthesized and real CT images.	China	2021	Elsevier	P32
Hsu, Ko-Tsung [68]	GAN	CT	Model-based learning outperforms other methods despite longer reconstruction times.	USA	2021	Elsevier	P33
Leuschner [69]	CNN	CT	Deep-learning-based methods consistently improved reconstruction quality metrics in both low-dose and sparse-angle CT applications.	Germany	2021	MDPI	P34
Matsuura M [70]	DLR	CT	The feature-aware DLR method outperforms conventional FBP and standard MBIR techniques in improving CT image quality.	Japan	2021	IEEE	P35
Capps M [71]	D-bar reconstruction	CT	The proposed approach is evaluated on simulated and experimental data representing the heart and lungs.	USA	2021	IEEE	P36
He J [72]	DSigNet	CT	Clinical patient data is used to demonstrate the effectiveness of DSigNet in achieving accurate CT image reconstruction.	China	2021	IEEE	P37
Ding Q [73]	CNN	CT	The effectiveness of the proposed method is evaluated using both simulated and real data.	Singapore	2021	IEEE	P38
Benz [74]	DLIR	CT	DLIR lowers CCTA radiation dose by 43% with minimal impact on accuracy.	Switzerland	2021	Springer	P39
Hammernik [75]	variational network	CT	The approach achieves superior destreaking results compared to existing non-linear filtering methods.	Austria	2021	Springer	P40
Noda [76]	DLIR	CT	DLIR improved image quality and reduced IC variability, suggesting its potential benefits for pancreatic dual-energy CT.	Japan	2021	Springer	P41
De Santis [77]	DLIR	CT	DLIR_M yields similar objective quality to ASiR-V 80% and 90%, excelling.	Italy	2021	Springer	P42
Kim [78]	DLIR	CT	DLIR at higher strength levels demonstrated reduced noise, and improved contrast-to-noise ratio compared to ASIR-V.	Republic of Korea	2021	Springer	P43
Thapaliya [79]	DLR	CT	All DLR algorithms showed substantial to almost perfect agreement on the presence of urinary tract calculi.	USA	2021	Springer	P44
Greffier [80]	AI-DLR	CT	The study found that using the Smooth and Smoother levels of the AI-DLR algorithm reduced image noise.	France	2021	Springer	P45
Kuo, C [81]	CNN	CT	Dice coefficient of 91.57%, a MioU of 89.43%, and a pixel accuracy of 99.75%.	Taiwan	2022	MDPI	P46
Lenfant [82]	DLR	CT	The effective dose decreased as the tube voltage decreased (1.5 mSv for 120 kVp, 1.1 mSv for 100 kVp, and 0.68 mSv for 80 kVp).	France	2022	MDPI	P47
Hu D [14]	DEER	CT	The DEER network’s performance is evaluated using a cone-beam breast CT dataset acquired from a commercial scanner.	USA	2022	IEEE	P48
Xie H [83]	PWLS	CT	The proposed method’s effectiveness is demonstrated using clinical SDCT and simulated LDCT scans from ten patients.	USA	2022	IEEE	P49
Park HS [84]	wGAN	CT	Machine learning approaches (wGAN and CNN) outperform FBP in image quality.	Austria	2022	Springer	P50
Thaler [85]	DDCNN, DnCNN, Win5RB	CT	U-Net best performance, with the lowest MAE, highest PSNR, and highest SSIM.	Japan	2022	Springer	P51
Koike [86]	DLIR, IR	CT	The use of DLIR significantly reduced image noise and improved image quality in pancreatic LDCT images compared to hybrid-IR.	Japan	2022	Springer	P52
Noda [87]	DLR, Hybrid-IR, MBIR	CT	In terms of image noise, LD DLR and LD MBIR images were superior to SD hybrid-IR images in the hepatic arterial and equilibrium phase.	Japan	2022	Springer	P53
Nakamura [88]	DLIR	CT	DLIR achieved comparable image quality in upper abdomen chest CT with <50% of the radiation dose.	Republic of Korea	2022	Springer	P54
Nam [89]	3D DPI	CT	Successful visualization of 3D alveolar units of intact mouse lungs at expiration and measurement of alveolar diameter.	Republic of Korea	2023	Nature Publishing Group UK London	P55
Shin [90]	DCNN	CT	The proposed method outperformed other 2D, 2.5D, and 3D methods in diagnosing drowning.	Japan	2023	IEEE	P56
Zeng Y [91]	VAE	CT	The model achieved a sensitivity of 79.2%, specificity of 72.7%, accuracy of 77.1%, F1-score of 0.667, and AUROC of 0.801.	Republic of Korea	2023	Nature Publishing Group UK London	P57
Chung [66]	CNN	CT	The developed deep learning network enabled high-accuracy estimation of 3D bone models.	Japan	2023	Springer	P58
Shiode [92]	DLR	CT	DLR showed finer image texture compared to one of the traditional methods and was closer to another in terms of texture.	France	2023	Springer	P59
Bornet [93]	DLR	CT	DLR led to significantly lower image noise and higher CNR compared to hybrid-IR and MBIR images.	Japan	2023	Springer	P60

**Table 4:** Overview of datasets available for training and validation in 3D deep learning for computed tomography reconstruction.

Author	Dataset	TEST SET	Population	Year	Database	Country	Ref.
Setio [41]	LIDC	118,650,898	CT	2016	IEEE	Germany	P01
Li, Meng [42]	LIDC	20,672	CT	2018	Springer	USA	P02
Wang [43]	Fudan University Shanghai Cancer Centre	200	HRCT	2018	pubmed	China	P03
Gruetze [44]	LUNA16	1186	CT	2018	https://academic.oup.com/	USA	P04
Gu, Yu [45]	LUNA16	1186	CT	2018	Elsevier	China	P05
Ren, Xuhua [46]	LUNA16	1186	CT	2018	Wiley Online Library	China	P06
Gupta H [47]	2016 NIH-AAPM-Mayo	500 CT images (1493 pixels in the view direction) (720 views)	CT	2018	IEEE	USA	P07
Li, Xiang [48]	Massachusetts General Hospital	200	CT	2019	Elsevier	Switzerland	P08
Uthoff [49]	INHALE study	100	CT	2019	Wiley Online Library	USA	P09
Annarumma [50]	Kings College London	15,887	X-rays	2019	RSNA	USA	P10
Lee H [51]	Evaluation utilized lung CT data from 8 distinct patients	662 slices	CT	2019	IEEE	UK	P11
Jung, Woojin [52]	k-space data	240 scans	MRI	2019	Springer	Republic of Korea	P12
Jiang, Chenyu [53]	carotid DECTA datasets	28 consecutive patients	CT	2019	Springer	China	P13
Sato, Mineka [54]	contrast-enhanced DECT images	40 patients	CT	2019	Springer	Japan	P14
Park, Sungeun [55]	not mentioned	CT images from 80 patients	CT	2019	Springer	-	P15
Higaki, Toru [38]	CMSC	CT images reconstructed with MBIR	CT	2020	Elsevier	Republic of Korea	P16
Singh, Satya P [20]	ADNI dataset	345 AD, NC, 605,991 MCI	MRI	2020	MDPI	Japan	P17
Zhang J [56]	HR (high-resolution) medical CT images	not mentioned	CT	2020	IEEE	China	P19
Liang, C-H [57]	Kaohsiung Veterans General Hospital, Taiwan	100	X-rays	2020	Elsevier	Taiwan	P20
Ichikawa [61]	Patients who underwent medical checkups	1831 chest and 519 abdominal CT scout images	CT	2020	Springer	Japan	P24
Oostveen [62]	not mentioned	50 consecutive patients	CT	2020	Springer	Netherlands	P25
Zeng [63]	Reconstructing raw data with FBP to obtain (SDCTTrain)	100,000 images	CT	2021	Elsevier	China	P27
Verhelst [64]	CBCT images	160 scans	CT	2021	Elsevier	Belgium	P28
Aggarwal [65]	not mentioned	not mentioned	CT	2021	Nature Publishing Group UK London	UK	P29
Han XF [120]	MSCT	not mentioned	CT	2021	IEEE	China	P30
Zeng [63]	DELTA	100,000 images	CT	2021	Elsevier	China	P31
Jiang, Hao [67]	not mentioned	888 lung cancer CT scans	CT	2021	Elsevier	China	P32
Hsu, [68]	not mentioned	(500) training, (500) testing	CT	2021	Elsevier	USA	P33
Leuschner [69]	LoDoPaB-CT dataset	40,000 scan slices from around 800 patients	CT	2021	MDPI	Germany	P34
Matsuura M [70]	not mentioned	5000 images	CT	2021	IEEE	Japan	P35
Capps M [71]	ACE1 EIT system at Colorado State University	100,000 scattering	CT	2021	IEEE	USA	P36
He J [72]	2016 NIH-AAPM-Mayo	4791 slices	CT	2021	IEEE	China	P37
Ding Q [73]	2016 NIH-AAPM-Mayo	2000 training epochs	CT	2021	IEEE	Singapore	P38
Benz [74]	TrueFidelity, GE Health-care	50 patients	CT	2021	Springer	Switzerland	P39
Hammernik [75]	not mentioned	450 fan-beam projections of size 512 × 512	CT	2021	Springer	Austria	P40
De Santis [77]	not mentioned	51 patients	CT	2021	Springer	Italy	P42
Kim [78]	not mentioned	62 patients underwent noncontrast brain CT scans	CT	2021	Springer	Republic of Korea	P43
Thapaliya [79]	not mentioned	14 patients, with a mean age of 17.3 years	CT	2021	Springer	USA	P44
Kuo, C [81]	not mentioned	75 datasets obtained from 111 men and 64 women	CT	2022	MDPI	Taiwan	P46
Hu D [121]	Koning Corporation	19,575 breast CT images from 42 patients	CT	2022	IEEE	USA	P48
Xie H [83]	2016 NIH-AAPM-Mayo	LDCT and SDCT images of size 512 × 512.	CT	2022	IEEE	USA	P49
Thaler [85]	not mentioned	13,650 slices	CT	2022	Springer	Japan	P51
Koike [86]	pancreatic ductal adenocarcinoma (PDAC)	28 consecutive patients	CT	2022	Springer	Japan	P52
Noda [87]	not mentioned	72 patients	CT	2022	Springer	Japan	P53
Nakamura [88]	not mentioned	100 patients	CT	2022	Springer	Republic of Korea	P54
Shin [90]	MSCT	not mentioned	CT	2023	IEEE	Japan	P56
Zeng Y [91]	not mentioned	334 CT images of normal orbits	CT	2023	Nature Publishing Group UK London	Republic of Korea	P57
Chung [66]	not mentioned	173 CT images, 105 X-ray images	CT	2023	Springer	Japan	P58
Bornet [93]	not mentioned	46 patients (December 2017 and April 2018)	CT	2023	Springer	Japan	P60

## Data Availability

Not applicable.
